# Development of a single device to quantify motor impairments of the elbow: proof of concept

**DOI:** 10.1186/s12984-022-01050-2

**Published:** 2022-07-21

**Authors:** Levinia Lara van der Velden, Bram Onneweer, Claudia Josephina Wilhelmina Haarman, Joyce Lisanne Benner, Marij Eugenie Roebroeck, Gerard Maria Ribbers, Ruud Willem Selles

**Affiliations:** 1 Department of Rehabilitation Medicine, Erasmus MC University Medical Center Rotterdam, Doctor Molewaterplein 40, 3015 GD Rotterdam, The Netherlands; 2 Rijndam Rehabilitation, Westersingel 300, 3015 LJ Rotterdam, The Netherlands; 3 Hankamp Rehab, Buurserstraat 198, 7544 RG Enschede, The Netherlands

**Keywords:** Spasticity, Synergy, Viscoelastic properties, Muscle strength, Mechanical device, Quantification, Stroke, Elbow, Upper limb

## Abstract

**Background:**

For patients with post-stroke upper limb impairments, the currently available clinical measurement instruments are inadequate for reliable quantification of multiple impairments, such as muscle weakness, abnormal synergy, changes in elastic joint properties and spasticity. Robotic devices to date have successfully achieved precise and accurate quantification but are often limited to the measurement of one or two impairments. Our primary aim is to develop a robotic device that can effectively quantify four main motor impairments of the elbow.

**Methods:**

The robotic device, Shoulder Elbow Perturbator, is a one-degree-of-freedom device that can simultaneously manipulate the elbow joint and support the (partial) weight of the human arm. Upper limb impairments of the elbow were quantified based on four experiments on the paretic arm in ten stroke patients (mean age 65 ± 10 yrs, 9 males, post-stroke) and the non-dominant arm in 20 healthy controls (mean age 65 ± 14 yrs, 6 males). The maximum strength of elbow flexor and elbow extensor muscles was measured isometrically at 90-degree elbow flexion. The maximal active extension angle of the elbow was measured under different arm weight support levels to assess abnormal synergy. Torque resistance was analyzed during a slow (6°/s) passive elbow rotation, where the elbow moved from the maximal flexion to maximal extension angle and back, to assess elastic joint properties. The torque profile was evaluated during fast (100°/s) passive extension rotation of the elbow to estimate spasticity.

**Results:**

The ten chronic stroke patients successfully completed the measurement protocol. The results showed impairment values outside the 10^th^ and 90^th^ percentile reference intervals of healthy controls. Individual patient profiles were determined and illustrated in a radar figure, to support clinicians in developing targeted treatment plans.

**Conclusion:**

The Shoulder Elbow Perturbator can effectively quantify the four most important impairments of the elbow in stroke patients and distinguish impairment scores of patients from healthy controls. These results are promising for objective and complete quantification of motor impairments of the elbow and monitoring patient prognosis. Our newly developed Shoulder Elbow Perturbator can therefore in the future be employed to evaluate treatment effects by comparing pre- and post-treatment assessments.

**Supplementary Information:**

The online version contains supplementary material available at 10.1186/s12984-022-01050-2.

## Background

Upper limb impairment following stroke significantly limits movement and obstructs functional performance of daily activities, such as reaching, grasping, and manipulating objects, and is classified into one or more of the following impairments: muscle weakness, sensory loss, stroke-related pain, abnormal synergy patterns, changes in viscoelastic muscle and joint properties, and spasticity [1]. To provide appropriate treatments and accurate prognosis that facilitate functional recovery, precise and quantitative description and classification of multiple upper limb impairments are essential [2, 3].

Multiple clinical instruments, such as the Brünnstrom Fugl-Meyer scale (BFM), Modified Ashworth Scale (MAS) and Modified Tardieu Scale (MTS), are currently used to quantify upper limb impairments [4, 5]. However, these instruments have several known limitations, including high inter-rater variability, poor validity, and lack of detailed information due to ordinal scaling. For example, in an earlier meta-analysis including 33 studies, fair to good inter-rater agreement was observed with the Modified Ashworth Scale [6], while validity, expressed as the relationship with electromyographic parameters, was only moderate [7, 8]. Based on its fair to good intraclass correlation coefficient (ICC) and poor relationship with electromyography, Fleuren et al*.* [9] concluded that the MAS should not be used in clinical practice to evaluate spasticity. Similarly, the Modified Tardieu Scale only had fair to good inter-rater agreement with an ICC of 0.58 [10]. Another problem of measurement of upper limb impairment is the lack of effective discrimination between muscle activity-based spasticity and viscoelastic joint properties [7, 8].

Several robotic quantification methods have been introduced to improve the currently available clinical measurement instruments for upper limb motor impairment [11–16]. The primary goal is reliable quantification of muscle weakness, abnormal synergy patterns, changes in viscoelastic joint properties and spasticity in a valid, responsive, and operator-independent manner [11–15]. Robotic devices generally act by imposing positions or forces on a joint and objectively quantifying patient response in terms of resistance or position. The majority of these devices have successfully achieved separate quantification of muscle activity-based spasticity and muscle and joint-related changes in viscoelastic properties of the hemiparetic arm. For example, Van der Krogt et al. [11] quantified spasticity by measuring the response of a patient's wrist to position-controlled tasks imposed by a robot. Additionally, McPherson et al*.* [16] quantified spasticity and changes in viscoelastic joint properties separately by applying different velocities to the limb with a position-controlled robot. Other groups have designed robotic devices to quantify abnormal synergy patterns. Ellis et al*.* [15] measured changes in the total reaching range of motion (work area) when the level of support provided to the arm was gradually lowered, where the reduction in the workspace with decreasing arm support was used as a measure of abnormal synergy.

A limitation of the previously mentioned robotic devices is that most of these devices measure only one or two upper limb impairments with a single device, preventing adequate study of their interactions and hampering clinical implementation. Here, we describe the design of a novel diagnostic robotic device designated the Shoulder Elbow Perturbator (SEP) and measurement protocol to quantify four important motor impairments: muscle weakness, abnormal synergy, changes in elastic joint properties and spasticity of the elbow in ten chronic stroke patients, compared to reference values of 20 healthy controls.

## Methods

### Participants

Ten chronic stroke patients from the outpatient clinic of Rijndam Rehabilitation Center were recruited. Patients that had suffered a stroke at least six months previously and with a documented upper limb impairment according to medical records were invited to participate in our study. Responders were screened based on the following inclusion criteria: self-reported upper limb impairment, ability to actively abduct the shoulder up to almost 80 degrees and visible active elbow extension, and minimal passive range-of-motion (ROM) in the shoulder joint of 0–80° abduction and 0–45° horizontal adduction. Patients were excluded in cases of hemiplegic shoulder pain, history of pre-existing neuromusculoskeletal disorders that could influence upper limb function, fixed contractures in the upper limb preventing test movements, or inability to understand instructions during the experiment. For comparison, a group of 20 age-matched healthy controls with no known history of neurological or orthopedic disorders was recruited. We elected not to use the contralateral arm as a comparison to reduce patient burden.

All participants provided written informed consent on the first day of the study prior to undertaking any study-related procedures. Our study was approved by the Medical Ethics Committee of the Erasmus University Medical Center Rotterdam.

### Shoulder elbow perturbator (SEP)

**Fig. 1:**
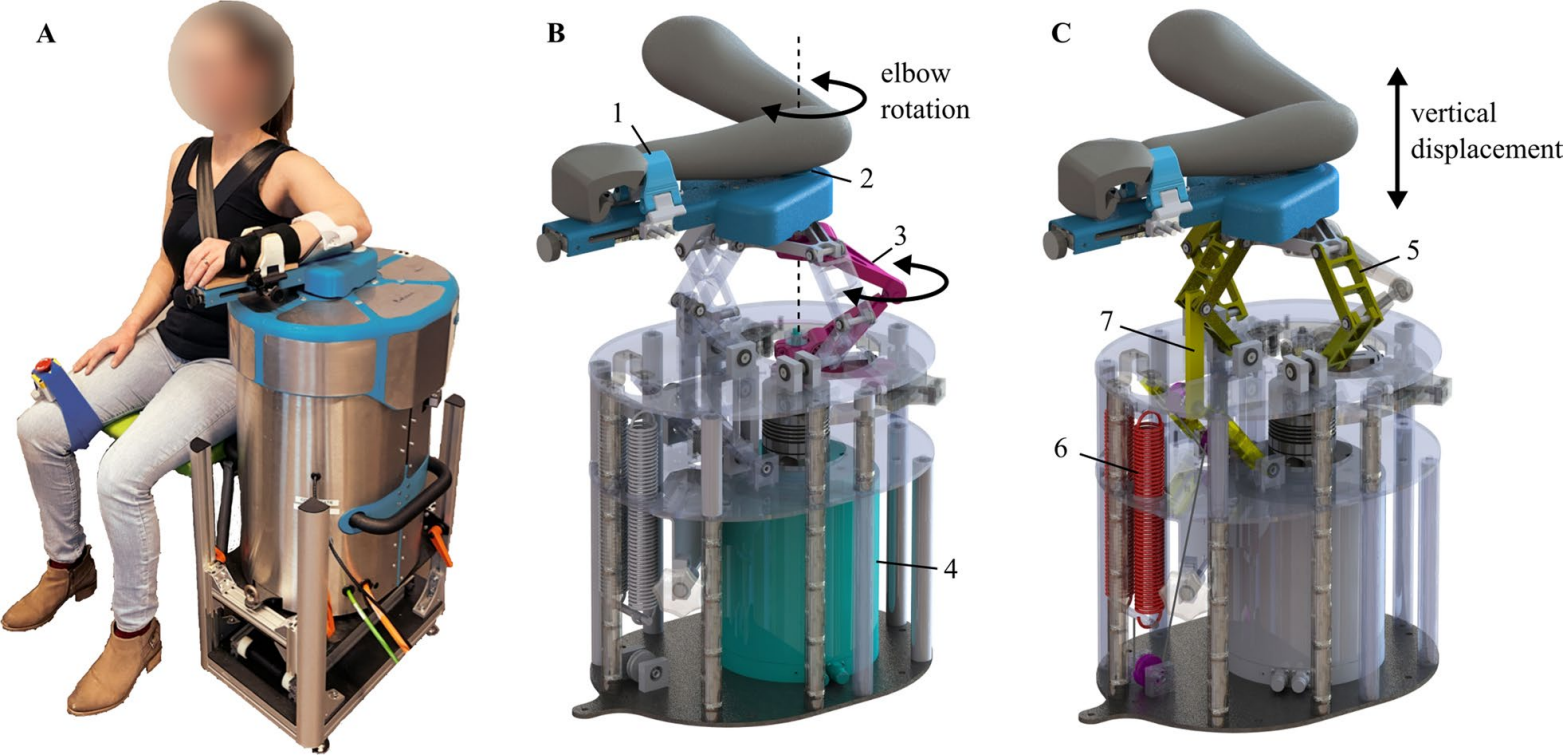
Overview of the Shoulder Elbow Perturbator (SEP), which manipulates shoulder abduction activity for elbow function quantification. **A** Participant seated in the SEP, strapped in the chair and with an emergency button attached to the upper leg. The forearm is fixated with the shoulder abducted in 80 degrees. **B** Internals of the SEP and illustration of how the wrist of the user is clamped (1) to the device with the elbow (2) aligned with the motor rotation axis. The torque link (3) transmits the torque of the motor (4) to the elbow. **C**) Internals of the SEP showing the sarrus and spring compensation mechanism. The sarrus linkages (5) allow for vertical displacement of the arm. The arm is supported by an upward force created by two springs (6). The cable routing and pulley configuration caused the upward force to be independent of the linkage position (7)

Data were collected with the SEP developed by Hankamp Rehab (Enschede, The Netherlands; Fig. 1). The SEP has an inertia of 0.014 kg/m^2^.The novel SEP could simultaneously manipulate the elbow joint angle (°) and support the (partial) weight of the human arm (steps of 25% total arm weight). A lever arm supported the human forearm. The elbow joint angle was controlled by a high-torque (180 Nm peak torque, 60 Nm continuous torque, 300 rotations/min nominal speed) direct-drive servo motor (‘HIWIN TMS3C’, Offenburg, Germany) aligned with the elbow (medial epicondyle of the humerus). A force sensor (LCM, Futek, USA, nonlinearity of ± 0.5%) positioned at a fixed distance between the rotational axis of the motor and lever arm measured elbow resistance (N) during rotation. The angular position of the lever arm was recorded by an optical incremental encoder (3600 lines/cycle, resolution of 0.25°/s) mounted on the motor axis. The forces (N) and angular positions (°) were converted to elbow torque (Nm) and elbow angle (°). The SEP was controlled from a PC using Etherlab and a custom Simulink model (The MathWorks Inc., Natick. Ma. USA). The torque, angular position, and motor input and output were saved with a sample rate of 1 kHz.

A torque link (Fig. 1B, pink) transmitted the torque of the motor (green) to the forearm to rotate the elbow. The sarrus linkage mechanism (Fig. 1C) allowed for vertical displacement. The (partial) weight of the arm was compensated with a passive spring mechanism (Fig. 1C). A cable attached to the spring (red) was routed over several pulleys such that the upward force on the elbow was independent of the mechanism height. This upward force could be adjusted by rotating a knob at the front of the SEP. Before each measurement, the desired upward force was verified by measuring the actual force with an HDB 10K10N (KERN & SOHN, Germany) hanging scale (10 kg weighing capacity, 50 g precisions and 10 g repeatability) mounted between the lever arm and base of the SEP.

### Participant safety

To ensure participant safety, several procedures were implemented. First, elbow rotation was restricted to the patient-specific passive range of motion with both mechanical end and software end stops. Second, when a torque above 66 Nm was registered (maximum limit), the motor was automatically turned off and the elbow could freely rotate. For fast elbow rotation, we arbitrarily downregulated the maximum torque limit to 11 Nm for safety reasons. Third, the participant could use an emergency button strapped around the leg to shut down the motor and allow free elbow movement. Fourth, wrist fixation had a quick-release system that released the wrist clamp from the SEP lever arm immediately by removing a pin.

### Experimental procedures

Participants were seated and strapped to the back of a custom-made chair with velcro straps crosswise over the shoulders to limit unwanted torso displacement (Fig. 1A). Each participant was positioned by adjusting the chair and SEP height manually so that the shoulder was abducted to 80º and horizontally adducted to 30º. The elbow (medial epicondyle of the humerus) of the participant was aligned with the rotator axis of the servo motor of the SEP and fixated with velcro straps. The wrist was positioned in ± 10º dorsiflexion with a cock-up cast and fixated by two clamps (Fig. 1A, B(1)).

### Measurement protocol

Prior to the experiments, age, sex, date and type of stroke, time post-stroke, hemiparetic arm and dominant arm were obtained from medical records, and body mass and length of participants was measured. The Modified Tardieu Scale was applied by moving the hemiparetic arm as slowly as possible (V1) for measuring the final elbow extension angle (R2) with a goniometer. Next, the hemiparetic arm was manually moved as rapidly as possible (V3) to measure the final elbow extension angle (R1). The quality and angle of muscle reaction (R2-R1) were registered [17].

**Fig. 2:**
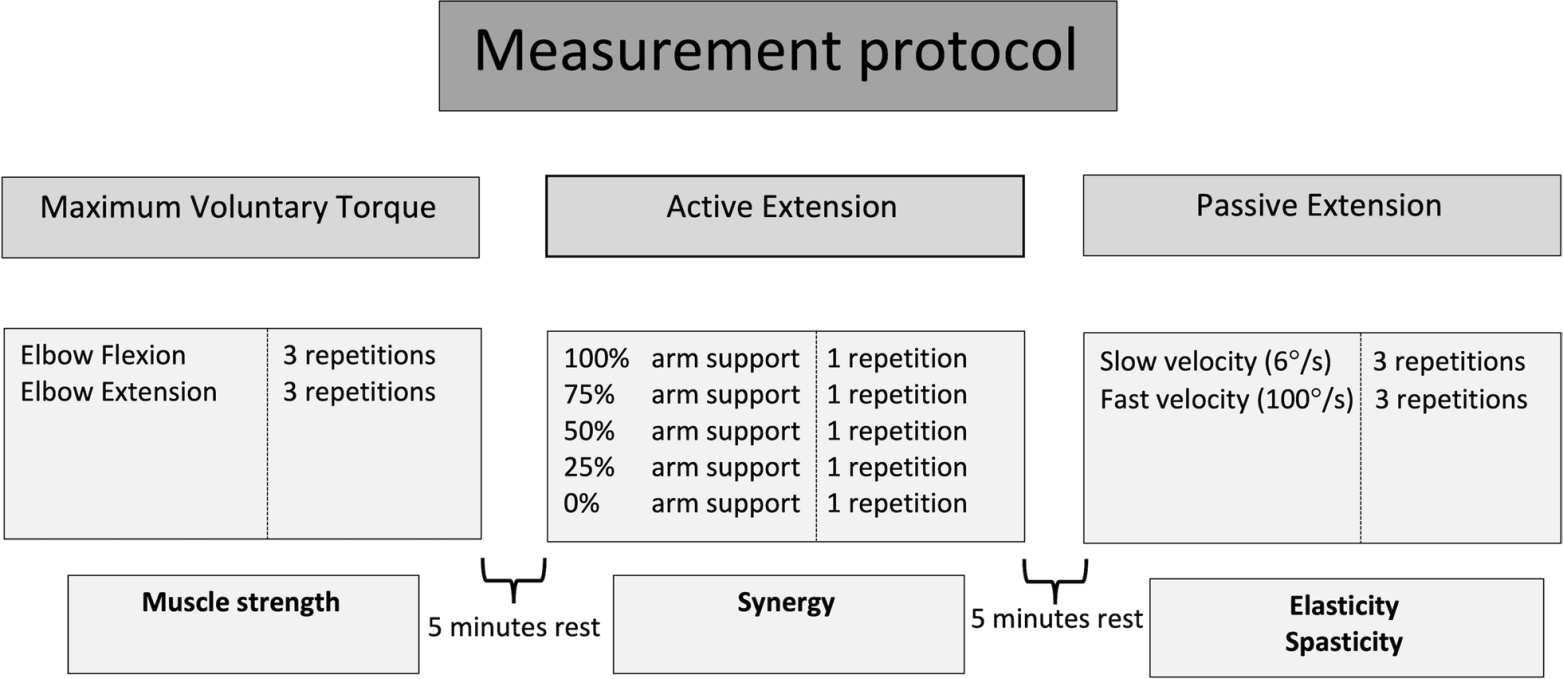
Measurement protocol. Three experiments were conducted to quantify muscle strength (elbow flexion and elbow extension strength), abnormal synergy, elasticity, and spasticity

An overview of the measurement protocol and impairments from each experiment is shown (Fig. 2). Three different experiments were performed to quantify muscle weakness, abnormal synergy, changes in elastic joint properties, and spasticity with the SEP. To prevent order and fatigue effects, experiments were presented in random order over participants. Before any experiment started, the participants arm was positioned in 90º elbow flexion and were asked to relax their arm; after that, the force sensor was calibrated to provide accurate measurements. The ‘Maximum Voluntary Torque’ experiment was employed to quantify muscle weakness of elbow extensors/flexors with full arm weight support in a fixed position of 90º elbow flexion. Participants were instructed first to relax their arm for 5 s to establish a resting torque, and subsequently, extend and flex their elbow to the maximum force in 5 s. Both flexion and extension directions were repeated three times, and in case a repetition deviated by ˃10% of the largest peak torque, the lowest peak torque was omitted and additional repetition was performed.

The ‘Active Extension’ experiment was performed to assess the amount of abnormal synergy, which represents reduction of the maximum elbow extension upon increase of shoulder abduction muscle activity. This experiment was inspired by previous experiments where either the active workspace of the arm [15] of the maximum extension strength [18] as a function of shoulder loading was used to quantify synergy. Participants started in the maximal elbow flexion mode and were asked to slowly (to minimize the velocity-dependent resistance and to eliminate the reflex response) extend the elbow as far as possible under randomly assigned different arm weight support levels (100%, 75%, 50%, 25%, and 0% arm weight support levels). The maximum elbow extension for each arm weight support level was used for data analysis.

The ‘Passive Extension’ experiment was used to assess changes in elastic joint properties and contribution of spasticity to movement resistance by applying two different elbow extension velocities (6 and 100º/s, with a velocity accuracy of 0.002º/s (± 0.01) and 1.4º/s (± 1.39)) three times each while asking participants to fully relax their arm. First, the limit of the passive range of elbow motion was defined by manually moving the arm to maximum flexion and extension positions. The SEP subsequently rotated the elbow with a constant velocity profile between these limits. To quantify changes in elastic joint properties, we imposed slow extension and flexion movements. For quantification of spasticity, a fast extension movement was used, after which the experimenter moved the joint back slowly to a flexion position. After each extension and flexion movement, a rest period of 5 s was applied. During the movement, the force sensor measured the resistance applied by the arm of the participant. In case a predefined maximum resistance (11 Nm) was reached before full passive ROM, the extension movement was stopped and held in position for 5 s. For changes in both elastic joint properties and spasticity measurements, the torque profiles over the ROM were used for data analysis. The inertial components of the SEP and human arm were not taken into account in the analysis because of their limited contribution during constant velocities.

The total protocol used a 1-min rest period between repetitions within an experiment and a 5 min rest period between experiments to prevent fatigue. Total time was recorded for the entire protocol, from entering the measurement room until leaving it. Also, the time for each individual experiment was registered. After each experiment, the perceived burden and pain was evaluated using a numeric rating scale from 0 to 10, with zero representing “no burden” or “no pain”. Comments from the participants on the burden and pain were noted.

### Data analysis

For all experiments, elbow torques were calculated by multiplying the measured force with the (fixed) distance between the force sensor and elbow joint.

**Fig. 3:**
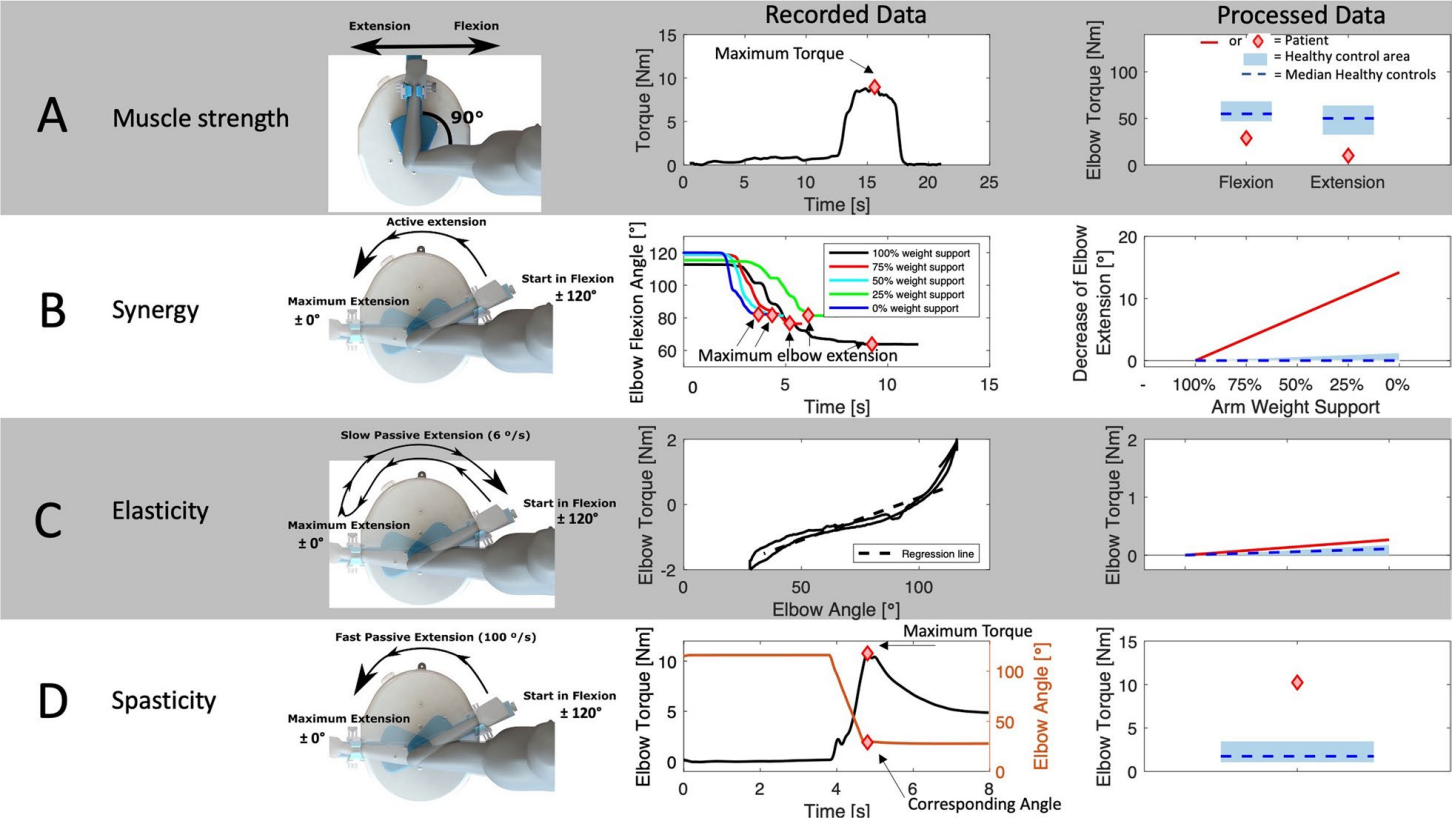
Illustration of each of the measurement protocols, includes a sketch of the participant's measurement assessment in the SEP, example recorded data of a stroke patient, and the processed data of the same stroke patient in comparison with the range of values of the healthy controls. **A** Muscle strength measurement, showing maximal isometric contraction in 90° elbow flexion for extension and flexion direction. Recorded data: Maximum torque (red diamond) during one torque profile of a stroke patient. We performed three repetitions, and maximum torque values were averaged. Processed Data: The flexion and extension torque of the stroke patient (red diamond) presented against the 10–90th percentile (blue area) of healthy controls. **B** Synergy, measured during an active extension movement of the elbow, repeated for five different arm weight support levels (100, 75, 50, 25, 0%). Recorded Data: Five position trajectories of the elbow extension with different arm weight support levels over time. The maximal extension angle (red diamond) of each trajectory was calculated, the elbow extension angle at 100% weight support was subtracted from each extension angle at 0, 25, 50, 75% arm weight support. Then, a regression line was fitted through these end positions and the slope of this regression line was used as an outcome parameter for synergy. Processed Data: The regression slope (red line) over de arm support levels for the stroke patients shown against in blue the 10-90th percentile of healthy controls. **C** Elasticity, measured during a slow (6°/s) passive movement from flexion to extension and back. Recorded data: A regression line fitted through the torque–angle trajectory of a stroke patient. We performed three repetitions, and the slope of the regression lines was averaged as outcome parameter. Processed Data: The regression line of the stroke patient (red line) shown against the 10-90th percentile of healthy controls (blue area). (**D**) Spasticity, measured during a fast (100°/s) passive movement trajectory from flexion to extension. Recorded data: The maximum resistance (red diamond) was calculated during the movement trajectory (orange line). We performed three repetitions, and the maximum torques were averaged as outcome parameter. Processed Data: The averaged maximum torque of the stroke patient was presented against 10-90th percentile of healthy controls (blue area)

For the ‘Maximum Voluntary Torque’ experiment, the calculated elbow torque was additionally filtered using a 250-point moving average filter to remove outliers. Next, the maximum torque for all repetitions was calculated and averaged for elbow flexion and extension as a parameter outcome for muscle strength (see Fig. 3).

For the ‘Active Extension’ experiment, the maximum elbow extension angle was calculated for each arm weight support level. The maximum elbow extension angle at 100% arm weight support was quantified as the maximum angle a patient maximally could achieve and, therefore, was taken to substrate this value from all extension angles so that the extension angle at 100% support was equal to 0. To relate the maximum angle to the weight support levels, the maximum elbow extension angle at 100% support was taken and was subtracted from each extension angle at 0, 25, 50 and 75% arm weight support level so that the extension angle at 100% was zero. A linear regression line was estimated through the extension angles at 0, 25, 50, 75 and 100% on the x-axis (interval of 25%). Since a decrease in extension angle with decreasing arm weight support indicates abnormal synergy, the slope of this regression line was used as a parameter for synergy (see Fig. 3).

From slow velocity movement during the ‘Passive Extension’ experiment, the mean torque at ten evenly spaced elbow positions was extracted during the slow flexion and extension movements. A linear regression line was fitted through these torque values and the three slopes were averaged and used as a parameter for elastic joint properties (see Fig. 3).

From fast velocity movement during the ‘Passive Extension’ experiment, the maximum torque applied by the participant during the predefined extension movement was averaged over the three repetitions as a parameter for spasticity (see Fig. 3).

Finally, for all impairments, we plotted data from each patient relative to the 10th percentile, median and 90th percentile of the control group. We used the 10th and 90th percentile and not the commonly-used 5th and 95th percentile because of the small sample size in this study. All impairments were subsequently combined in a radar chart for each patient to obtain an individualized upper limb impairment overview.

Since the main goal of this study was to perform proof-of-principle of the utility of the novel SEP in collective measurement of the four impairments, we selected a relatively heterogeneous group of participants and did not perform hypothesis testing.

## Results

### Participants

Ten chronic stroke patients with a mean age of 63 ± 11 years completed the measurement protocol between March 2019 and September 2019 (Table 1). Nine of these patients were male. Average age of stroke was 57 ± 9 years and average time post-stroke was 5 ± 3 years. The hemiparetic arm was dominant in four of the patients. The control group included twenty non-stroke aged-matched volunteers with a mean age of 57 ± 9 years, eight of whom were male.

**Table 1:** Characteristics of ten stroke patients and clinical test scores and perceived levels of pain and burden during the experiment

	Gender	Age (yr)	Time post-stroke (yr)	Type of lesion	Hemiparetic arm	Modified Tardieu Scale* (R2-R1 (quality score: (0–5))	Upper Limb Fugl-Meyer (Range 0–66)	Perceived pain during experiment** (0–10)	Patient-perceived burden of experiment** (0–10)
Patient 1	Male	73	4	Ischemic	Dominant	0 (0)	66	0	0.3
Patient 2	Male	69	7	Ischemic	Dominant	0 (0)	62	2.7	0.7
Patient 3	Male	68	8	Ischemic	Non-dominant	0 (0)	64	1.7	2
Patient 4	Male	66	7	Ischemic	Dominant	− 105 (2)	32	0	2.3
Patient 5	Male	50	5	Ischemic	Non-dominant	0 (1)	45	0	4
Patient 6	Female	73	4	Ischemic	Non-dominant	0 (1)	54	0	0
Patient 7	Male	76	9	Ischemic	Non-dominant	− 81 (2)	21	0.3	1
Patient 8	Male	51	1	Ischemic	Non-dominant	− 76 (2)	10	0	1.3
Patient 9	Male	47	2	Ischemic	Dominant	− 95 (3)	31	6.7	6.3
Patient 10	Male	54	1	Ischemic	Non-dominant	− 90 (2)	39	1.3	0.7

*Yr* years, *R2-R1* Angle of muscle reaction in degrees; Quality of muscle reaction (scale 0–5); *Range from 0–140 degrees **Averaged over all experiments for a patient

### Measurement duration and patient burden

The total measurement duration was 63 ± 11 min, including time for patient preparation and instructions, and MTS scoring. More specifically, the ‘passive extension’ experiment required 6 ± 2 min, ‘active extension’ 15 ± 3 min, and ‘maximum strength’ experiment 10 ± 3 min. The perceived burden of participation was scored 1.8 ± 2.3 (on a scale of 0 to 10) for stroke patients and 1 ± 1.1 for controls, with zero representing “no burden”. Pain for stroke patients was scored as 1.3 ± 2.2 (on a scale of 0 to 10) and controls scored 0.2 ± 0.5 (zero representing “no pain”). Patient 9 reported relatively high pain and perceived burden values (6.7 and 6.3 on a 0–10 scale) compared to the other participants. This patient arm function was very limited in function, and the experiments demanded much effort, especially activating the shoulder abductors to maintain the shoulder position during the active extension experiment. The prolonged shoulder position at 80 degrees caused a higher pain level, and when the arm returned to the neutral position (0° shoulder abduction), the pain disappeared immediately.

### Quantification of four upper limb impairments

**Fig. 4:**
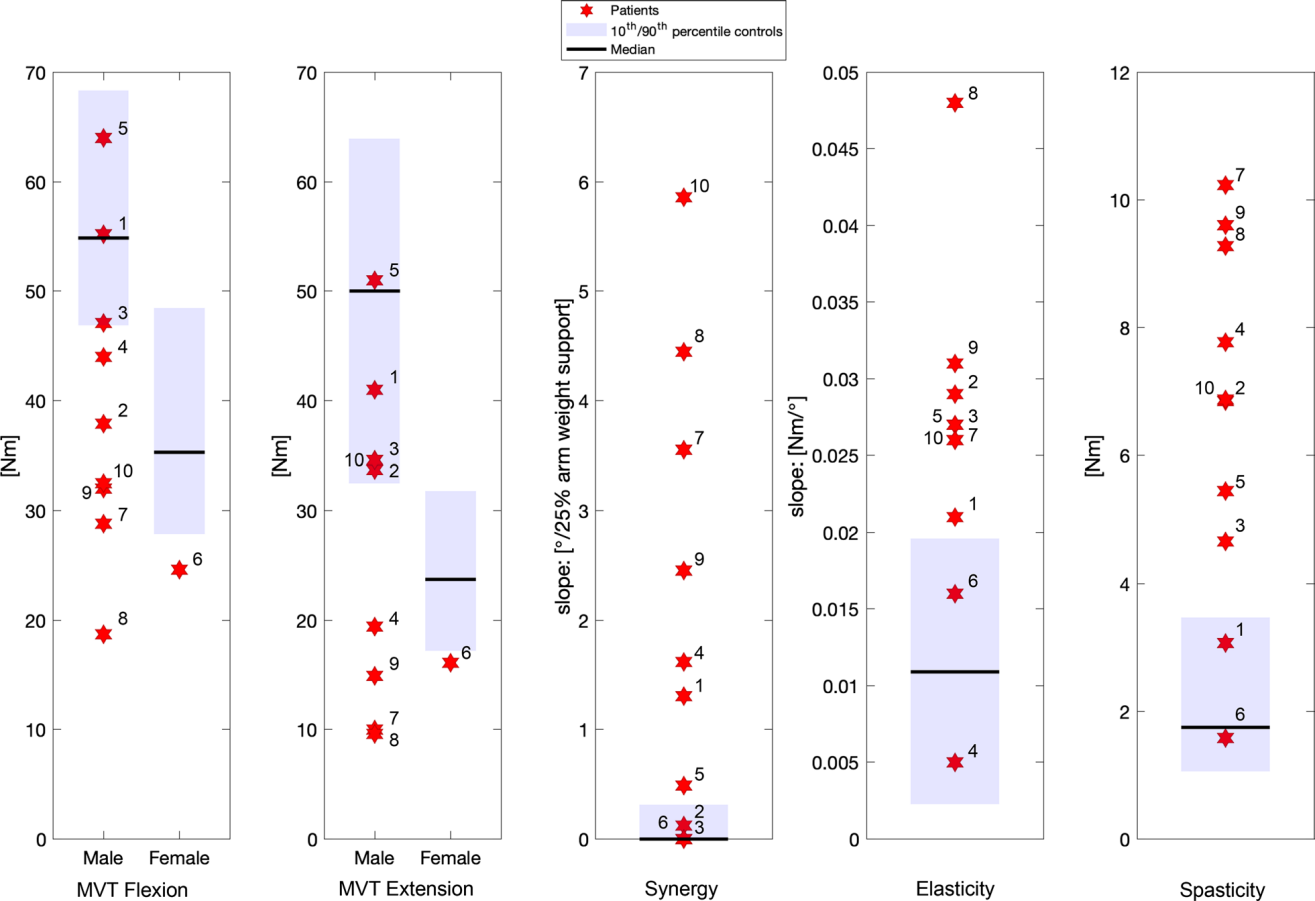
Results (red stars, numbers correspond to patient numbers in Table 1) for all motor impairments of the elbow of individual stroke patients. The 10th to 90th percentile reference interval of the controls is presented as a blue area. Measurement results for Maximum Voluntary Torque for flexion and extension (MVT flexion/ MVT extension) with a reference field specifically according to male and female controls. Measurement results for Synergy, Elasticity, and Spasticity

Impairment for all patients was visualized relative to 10th to 90th percentile reference intervals of the control group (Fig. 4), with patient numbers corresponding to the clinical characteristics described in Table 1. Five stroke patients showed muscle weakness in both directions (strength below 10^th^ percentile of the control group). Seven patients had synergy scores outside the reference interval of the control group, five with BFM < 40, while three patients with data comparable to controls had BFM > 40. Only two patients (1 and 5) had abnormal synergy patterns measured with the SEP but high BFM scores (> 40), indicating less synergistic movement pattern in the clinical test.

Elastic joint property values of eight stroke patients were outside the reference interval of the control group. For spasticity, eight stroke patients had increased maximum resistance torque during fast extension movement relative to the control group. Six of these patients had an abnormal MTS score (≥ 1, indicating resistance or a catch when the elbow is rapidly extended) and two stroke patients without increased maximum resistance torque had a normal MTS of 0 (Table 1). When evaluating all impairments, all patients showed at least one impairment value outside the reference interval of the control group. The recorded data and secondary measurement outcomes of all patients can be seen in the Additional file 1: Table S1.

### Patterns of upper limb impairment

**Fig. 5:**
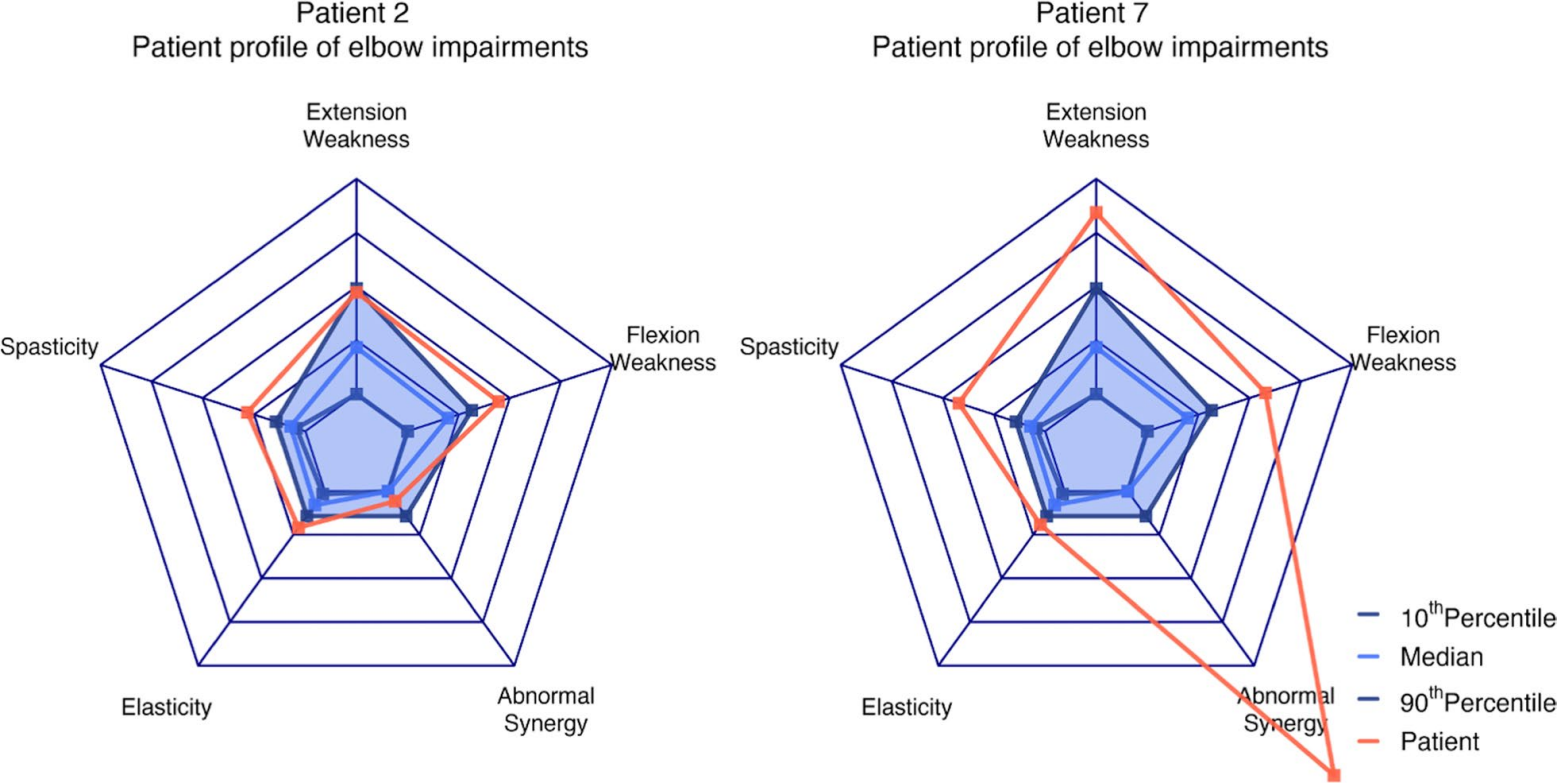
Two typical examples of radar charts visualization of all impairments in a single and interpretable graph. As a reference, median (light blue line) and 10–90th percentile reference intervals (dark blue line) of the controls are shown. The resulting blue area indicates no upper limb impairment. The values represent the extent of deviation of the patient (red point or line) from the 90th percentile. Both patients 2 and 7 had reduced flexion strength, slightly higher resistance during slow passive extension (Elasticity), and increased resistance during fast passive elbow extension (spasticity). Patient 7 also had reduced extension strength and maximum active elbow extension with decreasing arm support weight (abnormal synergy)

Figure 5 depicts two patients’ profiles (2 and 7) as typical examples to illustrate how SEP data can visualize different impairment combinations. In the profile of patient 2, only minor impairments in muscle strength, elastic joint properties, and spasticity were observed. In contrast, significant findings were obtained for patient 7, with a combination of loss of muscle strength for elbow extension and abnormal synergy pattern. All patient profiles are presented in Additional file 1: Fig. S1.

## Discussion

We have developed an innovative diagnostic device, the SEP, which could effectively differentiate and quantify muscle weakness, abnormal synergy, changes in elastic joint properties and spasticity of the elbow in a single session in stroke patients. Furthermore, patients perceived limited pain and low burden during these measurements. All impairments were visualized in a radar chart to facilitate clinical interpretation and implementation of appropriate therapeutic measures.

For specific impairments, our findings are in keeping with previously reported data on stroke patients obtained using robotic devices. Robotic devices, such as Biodex (ProCare, the Netherlands) are commonly used in clinical practice to determine muscle strength [19]. Our findings of reduced extension and flexion strength in stroke patients are consistent with previous reports on stroke patients [20–22]. For synergy, as expected, the maximum active elbow extension movement decreased when the arm weight support was decreased, indicating an abnormal flexion synergy pattern consistent with several previous studies reporting a linear relationship between increased shoulder abduction torque and decrease in the work area of the upper limb [1] [15] [20–24]. For elastic joint properties, we observed an increase in torque–angle responses during the slow passive movement of the elbow, keeping with studies by the research groups of Starky et al*.* [12] and Lorentzen et al*.* [25] using a similar method. In contrast, Given et al*.* [26] observed no increase in elasticity of the elbow in hemiparetic spastic stroke patients. In terms of quantification of spasticity, we observed higher maximum torque values in patients than healthy controls indicating increased resistance to fast velocities applied to the elbow, in accordance with other reports [27–29].

We used clinical instruments of spasticity (MTS) and synergy (BFM) as external criteria for quantitative SEP measurements in the absence of gold standards. In most cases, values recorded outside of the control range by the SEP were also reflected in the clinical scores. Further studies are essential to establish if the SEP indeed provides an assessment of the hemiparetic arm that is more reliable and discriminative than the commonly used clinical instruments with poor to moderate clinimetric properties.

To obtain insights into the extent of upper limb impairment, simultaneous evaluation of multiple impairments in one experiment is important [2]. The majority of documented literature to date has focused specifically on either changes in viscoelastic joint properties and spasticity [12, 13, 30, 31] or abnormal synergy and spasticity [32, 33], but limited research in kinetic measurement has evaluated multiple impairments recorded in the same patient. Since we performed all experiments with a single robotic device in the same participants, this study provides an overview of four important motor impairments associated with stroke within one patient and the creation of different patient profiles. For example, some patients (4, 7, 8, 9, and 10) showed both an abnormal synergy pattern and increased spasticity while abnormal synergy was not observed in other patients with spasticity (2, 5, and 3). Moreover, patients 7,8 and 9 had abnormal values in all 4 domains and patients 2 and 10 had decreased flexion strength with spasticity and normal extension strength. By presenting the impairment results in a radar chart with a reference area of aged-matched healthy controls, it is possible to effectively discriminate between healthy and impaired motor functions and degree of impairment, and thus compare outcomes within and between patients.

For this study, we made some trade-offs in the design of the device. Because we were interested in the impairments mentioned earlier, our measurement was limited to a single (elbow) joint movement. This does not reflect the whole hemiparetic arm function. Other robotic devices, such as the KinArm and InMotion, have made other design choices and are capable of measuring more joints and therefore also other variables, such as the reaching velocity or change in reaching distance due to stroke and caused by different sensorimotor impairments [34, 35]. However, these devices are not capable of varying the arm weight support to quantify abnormal synergy patterns. Moreover, the KinArm is not able to measure maximum muscle strength.

## Strengths and limitations

One major strength of this study is that four important domains of motor impairment can be measured in a single session. Combined recording of the different elbow impairments makes our device more clinically applicable compared to current devices that quantify only one impairment. Another strength is that the assessments are objective, quantitative, and more operator-independent than clinical instruments. In addition, the total measurement time is relatively short compared to other robotic protocols; all participants managed to execute the measurement protocol within 63 min (including patient preparation and instructions) on average, and could comfortably tolerate the measurement position and all experiments. A common problem is that robotic devices are more time consuming, but a clinician using clinical instruments to obtain the same measurements would need approximately 30 min to perform all the tests (± 5 min for MTS, ± 15 min for BFM and ± 8 min for muscle strength test with a dynamometer) [36–38]. However, the measurement time has not been described in most studies to date and may limit the clinical implementation of diagnostic devices [29].

The current study also has several limitations that need to be acknowledged. A major drawback is that the SEP cannot be used for all patients, since some severely affected stroke patients are unable to maintain the ± 80 degree shoulder abduction measurement position. In addition, although we attempted to fix the desired body position for each participant during the trials, some patients tried to compensate for inability to perform tasks by changing their body posture, thereby potentially causing small measurement deviations. Also, a limitation of the spasticity measurements was that inertial components from the weight of the SEP and human arm during acceleration and deceleration, as well as non-reflex properties were included in the maximal torque values within the fast ‘passive extension’ experiment. However, this effect is relatively small since the torque during the fast movement was so much larger than the torque values of elastic properties. In addition, the maximum torque as outcome of spasticity is insufficient to define the type of muscle activation, such as a clonus. Therefore, by suspicion of a clonus, in the future, the behavior of the torque–angle profile needs to be analyzed after a catch. Another limitation is the small sample size since this preliminary study was performed for initial evaluation of feasibility. Experiments with larger sample sizes are required to establish the reliability and validity of assessing upper limb impairments.

## Conclusions/clinical implications

In conclusion, we were able to quantify four important motor upper limb impairments objectively with the aid of a single device. Visualization of the four domains of upper limb impairments in a radar chart with a reference area of controls provides an easily interpretable overview of patient impairments, which is valuable for treatment planning and decision making by clinicians. Future studies may indicate the performance of the measurement protocol, the test–retest reliability and validity as well as the potential impact of this assessment for treatment selection in individual patients.

## Supplementary Information

**Additional file 1: Table S1.** Further details of the data recorded during the four SEP experiments. **Figure S1.** Radar charts for all patients visualizing all impairments in single and interpretable graph.

## Data Availability

The datasets used and/or analyzed during the current study are available from the corresponding author on reasonable request.

## Abbreviations

BFM: Brünnstrom Fugl-Meyer scale
ICC: Intraclass correlation coefficient
MAS: Modified Ashworth Scale
MTS: Modified Tardieu Scale
ROM: Range-of-Motion
SEP: Shoulder-Elbow-Perturbator
