# Supplementary material for: Development of a single device to quantify motor impairments of the elbow: proof of concept
Source: J Neuroeng Rehabil. 2022 Jul 21;19:77. doi: 10.1186/s12984-022-01050-2 (PMC9306071; doi:10.1186/s12984-022-01050-2)
Supplement: Supplementary file 1 — Additional file 1: Table S1. Further details of the data recorded during the four SEP experiments. Figure S1. Radar charts for all patients visualizing all impairments in single and interpretable graph. [file 12984_2022_1050_MOESM1_ESM.pdf]

Additional Table 1. Further details of the data recorded during the four SEP experiments. For the Maximum Voluntary Torque experiment, the three repetitions of maximum voluntary torque values are shown for flexion direction (1st column) and extension direction (2nd column). Active Extension experiment, the maximal elbow extension angle at five arm weight support levels is shown. Slow Passive extension experiment shows the starting passive flexion position and maximum passive extension position of the elbow in the SEP with the passive range of motion. Fast passive extension shows the time (1st column) and elbow angle (2nd column) where the maximum torque was calculated.

	**Maximum Voluntary Torque**		**Active Extension**	**Slow passive extension**	**Fast passive extension**	
	**Flexion: Maximum Torque for three repetitions [Nm]**	**Extension: Maximum Torque for three repetitions [Nm]**	**Maximal elbow extension angle [°]* for each arm weight support level (100%, 75%, 50%, 25%, 0%)**	**Passive range of motion [°]<br>Flexion – Extension (range)**	**Time of maximum Torque [s] for three repetitions**	**Elbow flexion angle [°]* at maximum Torque [Nm] for three repetitions**
Patient 1	53, 58, 55	39, 43, 41	9, 1, 2, 1, 2	130 – 13 (117)	1.2, 0.7, 0.9	12, 46, 13
Patient 2	33, 42, 40	29, 36, 36	12, 9, 10, 11, 9	126 – 11 (115)	1.2, 1.2, 1.2	10, 10, 11
Patient 3	47, 45, 50	35, 35, 34	12, 9, 9, 11, 11	127 – 18 (109)	1.1, 1.0, 1.0	26, 27, 52
Patient 4	46, 43, 42	19, 13, 20	7, 13, 14, 13, 17	115 – 20 (95)	1.0, 1.0, 0.9	21, 21, 29
Patient 5	59, 64, 65	47, 53, 53	7, 5, 6, 6, 5	115 – 17 (98)	0.5, 1.0, 1.0	69, 17, 18
Patient 6	22, 24, 27	14, 16, 18	19, 2, 3, 3, 5	121 – 4 (117)	1.2, 1.2, 1.2	-2, -1, 0
Patient 7	29, 29, 29	9, 11, 10	63, 76, 78, 81, 79	116 – 28 (88)	1.0, 0.9, 0.9	30, 29, 30
Patient 8	17, 22, 17	9, 9, 11	67, 80, 78, 81, 83	117 – 26 (91)	1.0, 0.9, 0.9	28, 27, 28
Patient 9	32, 31, 33	16, 13, 10	82, 93, 90, 101, 99	112 – 18 (94)	0.9, 1.0, 1.0	23, 19, 22
Patient 10	31, 32, 34	33, 36, 34	32, 29, 47, 46, 44	124 – 42 (82)	0.9, 0.9, 0.9	33, 33, 34

Note: * Elbow angles are noted in clinical perception evaluating the flexion angle of the elbow, with normative values healthy subjects elbow flexion ±150°, and normative values healthy subjects elbow extension ±0°.

Patient 1
Patient profile of elbow impairments

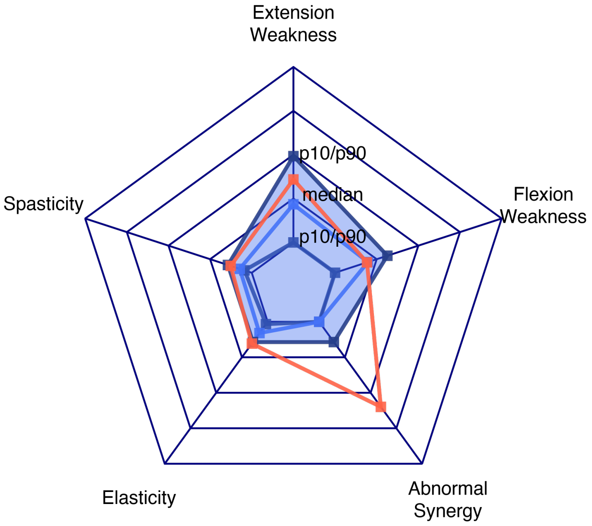

Patient 2
Patient profile of elbow impairments

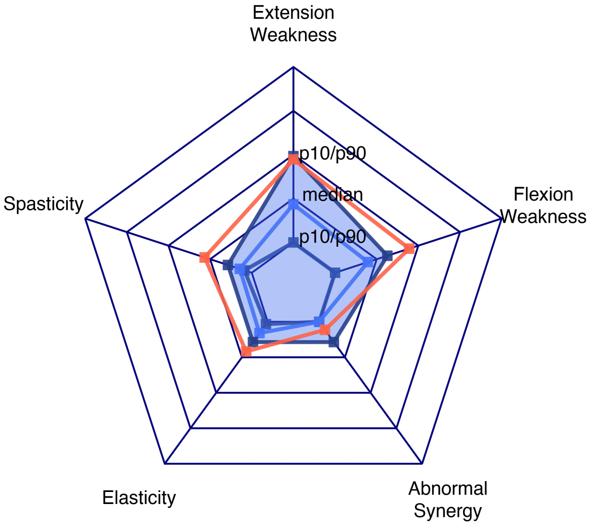

Patient 3
Patient profile of elbow impairments

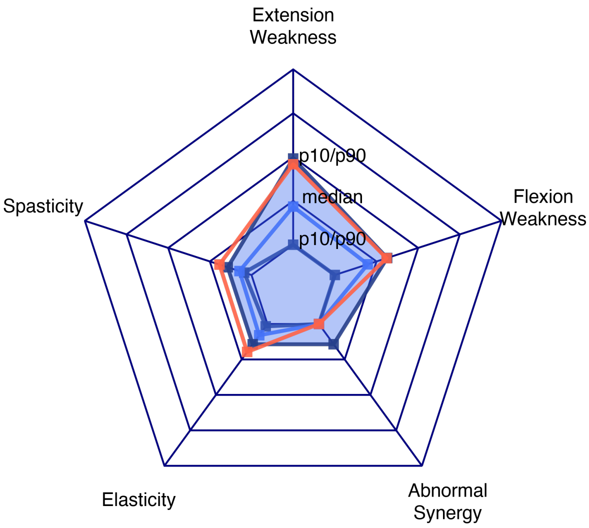

Patient 4
Patient profile of elbow impairments

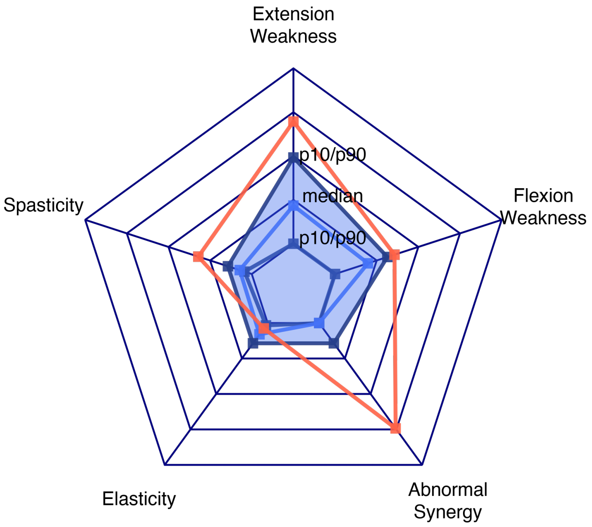

Patient 5
Patient profile of elbow impairments

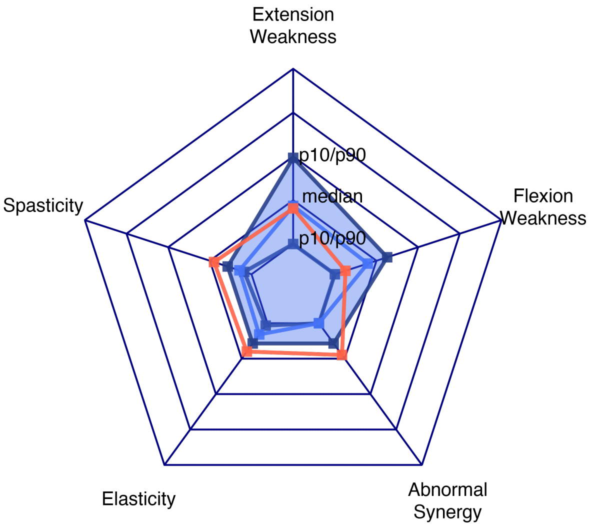

Patient 6
Patient profile of elbow impairments

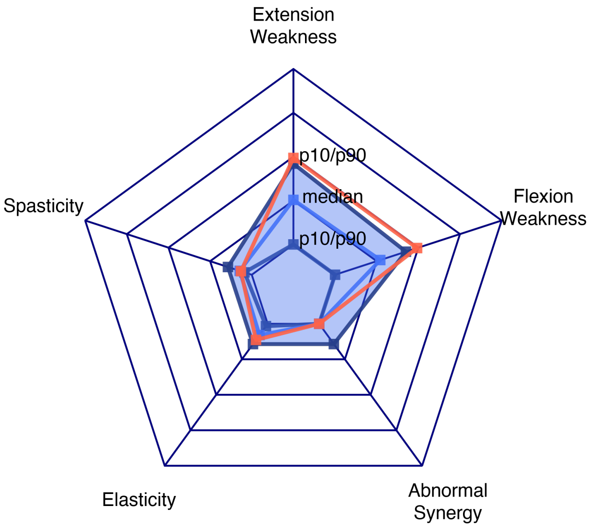

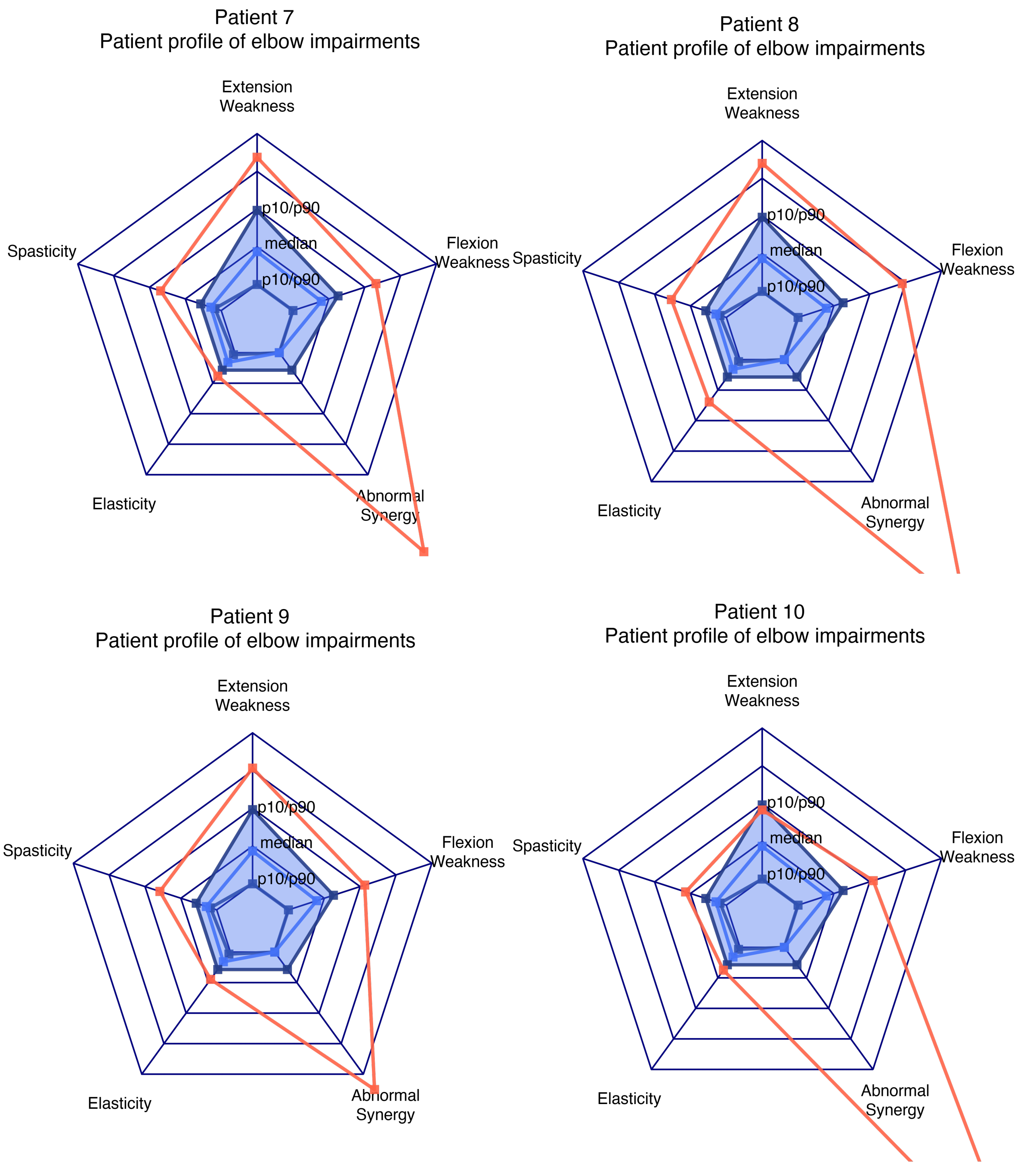

Additional Figure 1. Radar charts of all patients visualizing all impairments in a single and interpretable graph. As a reference, median (light blue line) and 10th-90th percentile reference intervals (dark blue line) of the controls are shown. The resulting blue area indicates no upper limb impairment. The values represent the deviation of the patient (red point or line) from the 90th percentile.
